# Cryo-EM structure of *P. falciparum* circumsporozoite protein with a vaccine-elicited antibody is stabilized by somatically mutated inter-Fab contacts

**DOI:** 10.1126/sciadv.aau8529

**Published:** 2018-10-10

**Authors:** David Oyen, Jonathan L. Torres, Christopher A. Cottrell, C. Richter King, Ian A. Wilson, Andrew B. Ward

**Affiliations:** 1 Department of Integrative Structural and Computational Biology, The Scripps Research Institute, La Jolla, CA 92037, USA.; 2 PATH’s Malaria Vaccine Initiative, PATH’s Center for Vaccine Innovation and Access, Washington, DC 20001, USA.; 3 The Skaggs Institute for Chemical Biology, The Scripps Research Institute, La Jolla, CA 92037, USA.

## Abstract

The circumsporozoite protein (CSP) on the surface of *Plasmodium falciparum* sporozoites is important for parasite development, motility, and host hepatocyte invasion. However, intrinsic disorder of the NANP repeat sequence in the central region of CSP has hindered its structural and functional characterization. Here, the cryo–electron microscopy structure at ~3.4-Å resolution of a recombinant shortened CSP construct with the variable domains (Fabs) of a highly protective monoclonal antibody reveals an extended spiral conformation of the central NANP repeat region surrounded by antibodies. This unusual structure appears to be stabilized and/or induced by interaction with an antibody where contacts between adjacent Fabs are somatically mutated and enhance the interaction. This maturation in non-antigen contact residues may be an effective mechanism for antibodies to target tandem repeat sequences and provide novel insights into malaria vaccine design.

## Teaser

An unprecedented spiral structure of the malarial CSP protein in complex with a protective antibody is revealed by cryo-EM.

## INTRODUCTION

**Fig. 1:**
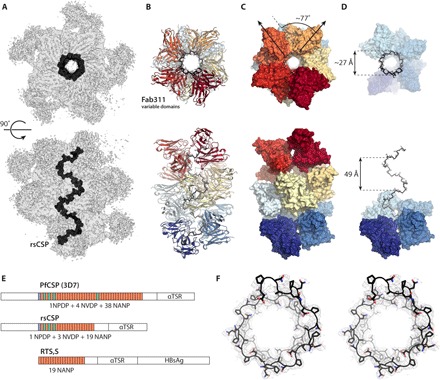
Spiral architecture of the rsCSP-Fab311 complex. (**A**) Side view and top view of the cryo-EM density map are shown as gray transparent surfaces. The density for the Fab constant domains is weak owing to flexibility around the elbow angle. rsCSP is colored black. (**B**) The variable domains for Fab311 are built in the cryo-EM map and shown in ribbon representation. The Fabs are colored ranging from dark red to dark blue. (**C**) The rsCSP-Fab311 structure is shown as a molecular surface to visualize the orientation of the Fabs. Each individual Fab variable domain is rotated ~77° with respect to the corresponding variable domain in a neighboring Fab. (**D**) Seven consecutive Fabs are stripped from the structure to unveil the buried repeat region of rsCSP that adopts a spiral. The pitch and diameter of the rsCSP spiral are 49 and ~27 Å, respectively. (**E**) Schematic diagrams of PfCSP, rsCSP, and RTS,S show the organization of the repeats within these three protein constructs. (**F**) Stereo representation of the rsCSP spiral (black carbons) and the cryo-EM density contoured at 5 σ as a gray mesh.

With an estimated 445,000 deaths and 216 million cases in 2016, malaria continues to pose a major threat to public health (*1*). Emerging resistance against current frontline antimalarials and insecticides has furthered the need for an efficient malaria vaccine candidate (*2*). The pre-erythrocytic stage of the *Plasmodium falciparum* life cycle is an ideal target for the development of a vaccine that disrupts the cycle of infection. After a bite from an infected mosquito, *P. falciparum* sporozoites migrate from the skin to the hepatocytes. Immunization with irradiated sporozoites can induce strong protective immune responses in mice, monkeys, and humans (*3*). For many years, the leading target for vaccine design has been the major surface protein of sporozoites, the *P. falciparum* circumsporozoite protein (PfCSP), which contains a central region consisting of multiple NANP repeats (*4*) that can vary (from 25 to 49) among different *P. falciparum* isolates (*5*, *6*). In addition, PfCSP contains a flexible N-terminal domain with a heparan sulfate binding site for hepatocyte attachment (*7*) and a structured C-terminal domain with a thrombospondin-like type I repeat (αTSR) (*8*). The most advanced malaria vaccine to date is RTS,S, formulated in GlaxoSmithKline’s adjuvant AS01. RTS,S contains part of PfCSP, including 19 NANP repeats and the αTSR domain, fused with hepatitis B surface antigen (HBsAg) (Fig. 1E) such that virus-like particles are formed when coexpressed with free HBsAg in yeast (*9*). The RTS,S vaccine has been shown to confer reasonable protection against clinical malaria in children (5 to 17 months old), with 51% protection over the first year of follow-up after a 0-, 1-, and 2-month vaccination schedule [95% confidence interval (CI), 48 to 55%]. Efficacy was seen to wane to 26% over a 48-month follow-up period (95% CI, 21 to 31%). If a boost is administered at month 20 after vaccination, efficacy is 39% (95% CI, 34 to 43%) (*10*–*12*). Long-term follow-up data up to 7 years after vaccination are now available (*13*) and indicate that, while the RTS,S vaccine is promising, an important objective in current malaria research is to improve and extend vaccine efficacy and durability. To date, very few attempts have been made to redesign or reformulate the current RTS,S vaccine. A promising effort is the R21 vaccine, which only differs from RTS,S in that it does not contain free HBsAg. The density of the CSP portion at the surface is therefore higher and presumably is a better mimic of PfCSP on the sporozoite surface (*14*).

One approach to improving vaccine designs involves structural investigation of monoclonal antibodies (mAbs) obtained through either whole sporozoite or RTS,S immunization. Recent x-ray structures of protective human Fabs in complex with PfCSP repeat peptides have revealed similarities and differences in how these repeats are recognized (*15*–*18*). Namely, the peptides are organized into NPNA structural units that can adopt type I β-turns and pseudo 3_10_ turns as originally observed for free peptides in solution and in peptide crystal structures (*19*, *20*). One of these antibodies, mAb311, was isolated from a phase 2a RTS,S/AS01B controlled human malaria infection (CHMI) clinical trial (*21*) and inhibited parasite development in the liver by ~97% as assessed by mouse challenge experiments with engineered *Plasmodium berghei* sporozoites that express PfCSP (*15*). A low-resolution, negative-stain electron microscopy (nsEM) reconstruction of a recombinant-shortened PfCSP construct (rsCSP; Fig. 1E) in complex with Fabs of mAb311 (Fab311) gave the first insight into organization of the NANP repeats with bound antibodies (*15*). However, a high-resolution structure would provide valuable information for optimal display of protective epitopes in a vaccine setting.

## RESULTS AND DISCUSSION

### Cryo-EM structure of CSP and architecture of the rsCSP-Fab311 complex

To decipher the architecture of the rsCSP-Fab311 complex at high resolution, we used single-particle cryo-EM. A final dataset of 206,991 particles was refined asymmetrically, resulting in an ~3.4-Å-resolution reconstruction (fig. S1). Eleven copies of the crystal structure of Fab311-(NPNA)_3_ could be fit into the EM map, and the rsCSP-peptide complex was then assembled in COOT to generate an initial model. This model was subjected to multiple rounds of refinement into the EM density map using RosettaRelax (Fig. 1A, figs. S1 and S2, and table S1).

**Fig. 2:**
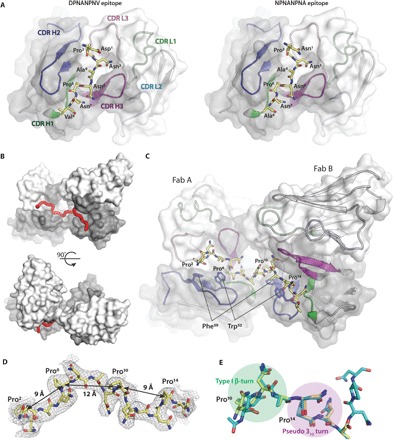
Epitope of two adjacent Fabs from the cryo-EM structure. (**A**) Illustration of the two different epitopes, DPNANPNV and (NPNA)_2_, that are observed in the cryo-EM rsCSP-Fab311 model. The heavy- and light-chain surfaces of the Fab variable domains are shown in gray and white, respectively. The 3D structures of the variable domains are shown in cartoon representation with complementarity-determining regions (CDR) H1, H2, H3, L1, L2, and L3 colored green, blue, magenta, light green, cyan, and pink, respectively. The rsCSP repeats are shown in stick representation (yellow carbons), and their amino acids are labeled and numbered from 1 to 8. (**B**) Front view and side view of the composite epitope on the variable domains of two Fabs, labeled A and B, that bind two adjacent (NPNA)_2_ repeats, with the tetrarepeat peptide shown as a red tube. (**C**) Detailed overview of the epitope shown in (B). The tetrarepeat (NPNA)_4_ is shown as sticks (yellow carbons), with the four prolines labeled Pro^2^, Pro^6^, Pro^10^, and Pro^14^ and with the (NPNA)_4_ repeats numbered 1 to 16. Hydrophobic residues—Trp^52^ and Phe^59^—that engage in CH/π interactions with the proline residues in the NPNA repeat are shown as sticks. (**D**) The cryo-EM density for the (NPNA)_4_ repeats is contoured at 5 σ and shown as a gray mesh. Distances between the Cα atoms of consecutive proline residues are highlighted. (**E**) Overlay of the peptide epitope from the cryo-EM structure (yellow carbons) with the corresponding peptide from the Fab311-(NPNA)_3_ crystal structure (teal carbons; Protein Data Bank: 6AXK) shows minimal differences. The type I β-turn and pseudo 3_10_ turn are highlighted by transparent green and magenta circles, respectively.

The repeat region of rsCSP is well defined with continuous cryo-EM density (Figs. 1F and 2D) and forms an unusual extended spiral structure (Fig. 1, A and D), from which multiple Fab311 antibodies radiate tangentially in a pseudo-helical arrangement (Fig. 1B), consistent with our previous nsEM reconstruction (*15*). In the cryo-EM map, however, two additional Fabs were observed, demonstrating that 11 Fabs can bind simultaneously to rsCSP (Fig. 1C), although the density for the N- and C-terminal Fabs was sparse. In addition, no density was observed for the N-terminal or C-terminal αTSR domains of rsCSP, likely due to flexibility. Although the αTSR domain has been observed to be structured by itself (*8*), it is connected to the NANP repeats through a disordered linker that is devoid of epitopes for Fab311. The angular twist between Fab variable domains is ~77° with respect to each other, where 4.7 Fabs (360°/77°) are required to complete one full turn of the spiral (Fig. 1C).

Helical conformations for NANP repeats have been proposed using computational methods. Gibson and Scheraga (*22*) used a modified buildup procedure to explore possible helical conformations for the (NANP)_6_ peptide, assuming that tandem repeats are likely to display helical or near-helical conformations driven by cooperative interactions. Two lowest-energy helices (left and right handed) were identified, with radii of 3.6 to 3.8 Å and a pitch of 7 and 10 Å, respectively. Brooks *et al.* (*23*) proposed an alternate, much wider, 12_38_ helix with a radius of 8.5 Å and a pitch of 4.95 Å using molecular dynamics (MD) calculations. Another model previously suggested a stem-like superhelix for the complete NANP repeat region, with a width of 15 Å (radius, 7.5 Å), a length of 180 Å, and a pitch of 7 NPNA repeats (*24*). Each of these helical predictions is very different from our structure. We observe a wider radius of 13.4 Å, a length of 145 Å, and a much larger pitch of 49 Å (9.5 NPNA repeats; Fig. 1, D and F). To complete a full turn on the spiral, a fifth Fab needs to partially pack underneath the first Fab, thereby making the pitch similar to the width of Fab311 along the longitudinal axis of the spiral (44.7 Å; ^L^Gly^68^ to ^H^Ser^74^ Cα-Cα distance). Our structure also differs from a recent model for an anti-NANP mouse antibody, 2A10, as a complex with NANP repeats. Here, antibodies are proposed to bind a narrow helix of repeats that adopt type I β-turns derived from MD simulations (*25*).

Because of the unique spiral architecture of rsCSP when bound to Fab311, we collected a cryo-EM dataset on another protective antibody, Fab317, in complex with rsCSP to improve the resolution of the previously published nsEM map, thereby gaining molecular details. Briefly, Fab317 was also isolated from the phase 2a RTS,S/AS01B CHMI clinical trial, had the same germline gene as mAb311 (VH3-33/30), and provided a 99.7% reduction of parasite liver load, as previously described (*15*). Fab317 binds up to three NPNA repeats in comparison to Fab311, which only requires two repeats. However, inspection of the two-dimensional (2D) class averages revealed various stoichiometries similar to those of the nsEM 2D classes, with up to five Fab317s bound to rsCSP. Although the dataset was subject to extensive rounds of computational processing and 3D sorting, reconstruction of the particles was unable to converge to high resolution (fig. S3).

### Fab311 epitope on rsCSP

Traditionally, the repeat region of PfCSP has been described by the number of NANP repeats. However, nuclear magnetic resonance and x-ray crystallographic evidence show that the repeats are likely organized as NPNA structural motifs (*15*, *19*, *20*). Hence, we adopt the NPNA nomenclature when discussing the epitope instead of the more general NANP notation. The Fab311 epitope was proposed to consist of a minimum of two to three NPNA repeats on the basis of the crystal structure of Fab311 with the (NPNA)_3_ peptide and isothermal titration calorimetry (ITC) affinity measurements (*15*). Here, the cryo-EM structure determines unambiguously that the epitope consists of only two NPNA repeats. The Fabs are so closely packed against one another that their two epitopes are seamlessly stitched together without the need of an additional repeat as a spacer. Furthermore, we observed that Fab311 is able to bind the NVDP repeats, thereby increasing the available epitopes on rsCSP from 15 (NPNA only) to 22 (including the DPNA and NPNV repeats). The only two sequence differences in DPNANPNV from NPNANPNA occur on the edge of the epitope and thus are likely minimally inhibitory to Fab311 binding (Fig. 2A). The Asp at the N terminus is in a similar conformation to the Asn, and the Val projects out into solvent.

The calculated BSA when taking two adjacent Fabs as one binding unit is 972 Å^2^ on the Fabs and 843 Å^2^ on the (NPNA)_4_ peptide. Fabs are positioned such that the groove in which the peptide resides extends from one Fab directly into the other (Fig. 2, B and C). Overall, there is excellent agreement with the epitope in the cryo-EM structure with the first two of three NPNA repeats observed in the crystal structure (Fig. 2E). The two repeats of the NPNA epitope adopt a type I β-turn followed by a pseudo 3_10_ turn (Fig. 2E) that repeats throughout the length of the spiral structure. Each pseudo 3_10_ turn has its asparagine (i) side-chain hydrogen bonding with the backbone amide of the next asparagine (i + 2). Because of this unique repetition of the (NPNA)_2_ epitope in rsCSP, the proline residues consistently point away from the center of the spiral, serving as anchor points to which the Fabs latch on (Fig. 1F). CH/π interactions of the prolines with ^H^Trp^52^ and ^H^Phe^59^ alternate (Fig. 2C), with Cα-Cα distances of 9 and 12 Å between each consecutive proline pair (Fig. 2D). ^H^Trp^52^ provides key contacts with the peptide and may account for the frequent selection of germline VH3-33 (and related VH3-30) for recognition of the NANP repeats (*17*, *18*, *26*).

### Inter-Fab contacts stabilize the CSP spiral structure

**Fig. 3:**
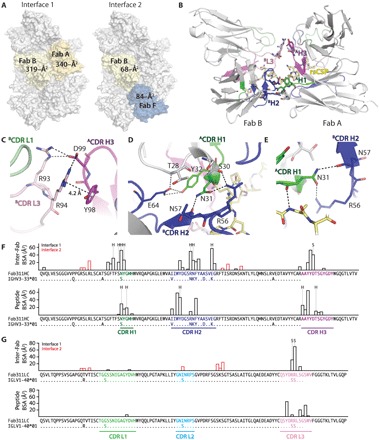
Somatically mutated inter-Fab contacts. (**A**) Surface representation of the rsCSP-Fab311 cryo-EM structure. Only the variable domains of Fab311 are shown. The Fabs of interest for which their mutual buried surface area (BSA) was calculated are colored yellow, orange, or blue, while other Fabs are colored gray. (**B**) Interactions between two Fabs (A and B) that bind adjacent epitopes on rsCSP. The Fabs are shown in cartoon representation with their heavy and light chains colored darker gray and lighter gray, respectively, and their CDR H1, H2, H3, L1, L2, and L3 colored green, blue, magenta, light green, cyan, and pink, respectively. The tetra repeat (NPNA)_4_ is colored yellow and shown in stick representation. The CDR loops that engage in inter-Fab contacts are highlighted. (**C**) Salt bridges in interface 2 between Arg^93^ and Arg^94^ of ^B^CDR L3 (pink) and Asp^99^ of ^A^CDR H3 (magenta) and a cation-π interaction between Arg^94^ of ^B^CDR L3 and Tyr^98^ of ^A^CDR H3 are shown as black dashed lines. Tyr^98^ in V_H_ is also shown because of its large BSA contribution. (**D**) Hydrogen bonding network (black dashed lines) in interface 1 between residues of ^B^CDR H2 and ^A^CDR H1. (**E**) Asn^31^ of ^A^CDR H1 hydrogen bonds with Ala^8^ of (NPNA)_4_ using its main-chain carbonyl (same numbering as Fig. 2C), while its side-chain hydrogen bonds with the backbone of Asn^57^ and the side chain of Arg^56^ of ^B^CDR H2 from a neighboring Fab. (**F** and **G**) Individual residue contributions to the BSA of inter-Fab and to the peptide repeat contacts are shown in a bar plot for the heavy chain (F) and light chain (G). The CDRs as defined by Kabat are colored as in the previous figures. In addition, somatically mutated residues in V_H_ and V_L_ are shown by the alignment of the Fab311 sequence with the germline V_H_ and V_L_ gene sequences (excluding CDR H3). Residues that engage in hydrogen bonding and salt bridges are marked with “H” or “S,” respectively.

It is unlikely that free PfCSP is predominantly present as a well-defined spiral on the surface of the sporozoite, since the repeat region is predicted to be disordered (*27*), and atomic force microscopy and single-molecule force microscopy experiments indicate that PfCSP can adopt multiple conformations (*28*, *29*). Thus, binding of Fab311 may induce and stabilize the rigid spiral structure in the NANP repeat region of PfCSP. Unexpectedly, neighboring Fabs that bind adjacent (NPNA)_2_ epitopes contribute 319- and 340-Å^2^ BSA to a novel interface between the Fabs (Fig. 3, A and B). Taking into account these additional contacts, the total BSA on each Fab with rsCSP and neighboring Fabs becomes 1145 Å^2^ [(972 Å^2^/2) + 319 Å^2^ + 340 Å^2^], which increases the original Fab-peptide BSA more than twofold. Close inspection reveals that the inter-Fab BSA between two Fabs (A and B) binding successive epitopes of the rsCSP (interface 1) spiral consists of polar contacts that are made between ^B^CDR L3/^A^CDR H3 and ^B^CDR H2/^A^CDR H1 (Fig. 3, B to D). Many residues that are involved in inter-Fab contacts correlate with somatic hypermutations from the IGHV3-33*01 and IGLV1-40*01 germline genes for the heavy and light chain, respectively (Fig. 3, F and G). Salt bridges are made between Asp^99^ of ^A^CDR H3 and Arg^93^ and Arg^94^ of ^B^CDR L3; in addition, a cation-π interaction is found between Arg^94^ of ^B^CDR L3 and Tyr^98^ of ^A^CDR H3, where Arg^94^ Nε and the center of the aromatic tyrosine ring are 4.2 Å apart (Fig. 3C). Furthermore, Asn^31^ of ^A^CDR H1 and Arg^56^, Asn^57^, and Glu^64^ of ^B^CDR H2 form an extensive hydrogen bonding network, which would be abrogated if reverted to the germline sequence (Fig. 3D). Most of these residues do not contact the NPNA repeat motifs, except for Asn^31^. The affinity maturation of inter-Fab contacts is likely driven by somatic hypermutation of Ser^31^ to Asn^31^, since Asn^31^ hydrogen bonds with the repeats using its main-chain atoms, while simultaneously forming a hydrogen bond with a neighboring Fab using its side chain (Fig. 3E). Other Fabs in close proximity are those that bind four epitopes away (B and F) such that they complete a full spiral turn and are either above or underneath the Fab of interest. Although some BSA is present between these two Fabs (interface 2), there are no direct contacts as assessed by CONTACSYM (Fig. 3A).

### Mutagenesis of the Fab311 interface

**Fig. 4:**
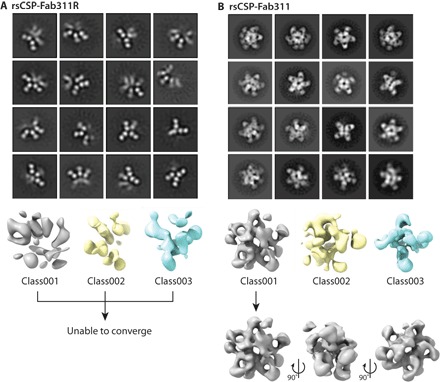
Germline reversion of inter-Fab contact residues. Representative class averages for (**A**) the germline-reverted Fab311 (Fab311R) in complex with rsCSP versus (**B**) somatically mutated Fab311 in complex with rsCSP (*15*). 3D classes of rsCSP-Fab311R (*1*–*3*) did not converge during refinement, while class001 of the rsCSP-Fab311 complex converged and could be further refined.

To investigate the specificity of the interactions between adjacent Fabs, somatically mutated residues that engage with neighboring Fabs were mutated to the inferred germline sequence (Fab311 inter-Fab contact residue reverted, Fab311R). Specifically, four and two residues were mutated in the heavy (N31S, R56N, N57K, and E64K) and light (R93S and R94S) chains, respectively. First, we assessed whether Fab311R can still bind to the (NPNA)_2_ peptide using ITC affinity measurements (fig. S4, table S2) and found that its binding is unperturbed, indicating that few, if any, mutations are required for high-affinity peptide binding. Next, we determined whether the germline reversion mutagenesis abrogated formation of the rsCSP spiral using nsEM. Unexpectedly, the 2D class averages revealed a new phenotype with varying stoichiometries for the rsCSP-Fab311R complex in which a well-defined long-range spiral was absent. Nevertheless, the rsCSP-Fab311R particles still adopted curved conformations in which the Fabs can still bind relatively closely together, indicating that some form of inter-Fab contacts may be encoded in the germline (Fig. 4). This heterogeneity led to the inability of the particles to converge into a stable 3D reconstruction, which could not be further refined. By comparison, 2D class averages of the wild-type rsCSP-Fab311 complex show a much more homogeneous and compact complex, providing further evidence that the somatically mutated inter-Fab residues play a crucial role in stabilizing the spiral architecture of rsCSP and presumably help gain increased avidity to CSP.

### Conservation of the spiral architecture

**Fig. 5:**
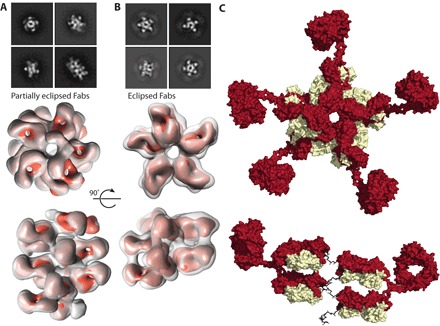
Helical architecture of full-length CSP with Fab311 and rsCSP in the presence of IgG311. Select reference-free 2D class averages and corresponding number of 30-Å low-pass–filtered Fab311s (orange) were docked into the nsEM 3D reconstructions of (**A**) flCSP in complex with Fab311 or (**B**) rsCSP in complex with IgG311. In both instances, CSP remains helical, with one complete turn equaling five epitopes. Ten Fabs (five IgG311s) can bind to rsCSP, with the Fabs assigned to one IgG molecule eclipsing each other (when viewed down the helical axis) compared with the partially eclipsed orientation of the Fabs in the flCSP-Fab311 complex. (**C**) A model of the rsCSP-IgG311 complex shows that the Fabs of IgG311 bind rsCSP parallel to each other, above and below each other on the spiral, but the Fabs from one IgG are separated on a linear scale along the spiral by four intervening Fabs. The Fc region was added to these two Fabs, and the structure energy was minimized with Rosetta.

To explore the physiological relevance of the spiral structure, we expressed full-length PfCSP (flCSP, based on the 3D7 strain) for nsEM studies with Fab311. The amino acid sequence of flCSP is identical to that of rsCSP, with the exception of the repeat region, which has 38 NANP repeats and 4 NVDP repeats, of which 3 are located at the N terminus and 1 is located in the middle of the NANP repeat region (Fig. 1E). The 3D reconstruction of flCSP-Fab311 revealed an identical helical architecture to the rsCSP-Fab311 complex (Fig. 5A and figs. S5 and S6). Since the number of NANP repeats is doubled in flCSP compared with rsCSP, we were expecting >20 bound Fabs. However, the total Fab count in the flCSP-Fab311 complex is only 14. One possible explanation is that the additional NVDP repeat in the center of the NANP repeat region breaks up the NPNA registry and rigidity of the structure, since the affinity for NVDP is approximately fivefold less than that for NANP repeats (*15*). Nonetheless, these results provide evidence that the spiral architecture can also be formed by PfCSP with a much larger number of repeats.

To answer the question of whether an individual immunoglobulin G (IgG) is capable of binding to two epitopes within the same rsCSP molecule and further stabilizing the spiral, we prepared an rsCSP-IgG311 complex for nsEM studies. A significant amount of aggregation was observed upon addition of IgG311 to rsCSP as a result of cross-linking rsCSP molecules, which has also been termed the CSP reaction (*30*). After removal of aggregates by spin filtration and subsequent size exclusion chromatography, we were able to separate the sample into soluble aggregates, the rsCSP-IgG311 complex, and unbound IgG311 fractions (fig. S5). In the nsEM 2D classes of the rsCSP-IgG311 complex, the Fc domains appeared as diffuse densities radiating from the Fabs that did not converge in the 3D reconstruction (Fig. 5B). The 3D reconstruction closely matched the nsEM map of rsCSP-Fab311, but with a subtle difference in the helical twist. Comparison of the top views of the two reconstructions shows that Fab311 binds rsCSP in partially eclipsed orientations along the length of the spiral, while the two Fab domains of each bound IgG311 lie on top of one another (Fig. 5, A and B, and figs. S5 and S6). Notwithstanding, the rsCSP still adopts a spiral structure of identical radius with the IgG, despite the additional geometric constraints that the hinge region of the IgG311 poses on binding. A total of five IgGs (10 Fabs) were bound to rsCSP, in comparison to only nine Fab311 in the nsEM 3D reconstruction (*15*). Thus, although IgG311 likely cross-links PfCSP on the surface of sporozoites, analysis of this minor population of single particles indicates that, just as for Fab311, IgG311 can bind with its Fab arms closely together and then still accommodate inter-Fab domain contacts between two different IgG molecules. The two Fabs that contribute to one IgG are then oriented such that the heavy and light chains are arranged light-heavy–light-heavy and are not symmetric (light-heavy–heavy-light), as depicted in cartoons in most textbooks (Fig. 5C).

### Structural ramifications and implications for vaccine design

The cryo-EM reconstruction of rsCSP saturated with Fab311 at 3.4-Å resolution demonstrates an unprecedented open spiral structure of rsCSP, which is still present with IgG or with flCSP. This structure differs substantially from previous predicted helical models for the NANP repeat region. Unexpectedly, the Fab domains make specific interactions not only with the NANP repeat region but also with neighboring Fabs along the NANP spiral surface. These inter-Fab contact residues have undergone somatic hypermutation and are crucial for spiral formation. This finding provides strong evidence for antigen-induced maturation of inter-Fab interactions for human antibodies, which may prove to be a common mechanism for increasing affinity against the PfCSP repeat region and for tandem repeat sequences in general. Recently, heavy-chain antibody fragments (nanobodies) derived from alpacas against a pentameric antigen were observed to have inter-nanobody contacts, suggesting that this mechanism may be present across certain antibodies in different species (*31*). A previous structure of antibody 2G12 to HIV Env revealed a novel domain swap within the Fabs of a single IgG molecule, where the heavy chain from one Fab paired with the light chain of the other Fab, such that a new V_H_-V_H_ interface was formed that was also subject to somatic hypermutation (*32*). However, that configuration differs from the Fab arrangement here, where we observe instead interactions between the Fabs that are connected to different IgG molecules. We do not know whether spiral formation correlates with protection, since mAb317 is bound in a less regular way to rsCSP, while being of similar efficiency to mAb311 in reducing the parasite liver load in mice experiments (fig. S3) (*15*). However, mAb317 2D class averages of the Fab bound to rsCSP are topologically similar to the more ordered mAb311 classes. Thus, it is likely that parts of the spiral may be present in the PfCSP conformational ensemble, perhaps even in the form of successive type I β-turns and pseudo 3_10_ turns, which, in effect, may code for the spiral preference in the presence of Fab311. If protection is correlated with affinity to the PfCSP repeat region (*21*), inter-Fab maturation and spiral formation could lead to more protective antimalaria antibodies.

A recently isolated human antibody (MGG4) in complex with the N-terminal junction peptide (KQPADGNPDPNANP) showed binding to the NPDP repeat in the junction region just before the repeat region (*17*), to which Fab311 is also capable of binding based on our cryo-EM structure. In addition, Fab311 and MGG4 have identical heavy-chain germline gene (VH3-33/30) usage and mode of binding through CH/π interactions between a proline in a pseudo 3_10_ turn and the conserved Trp^52^ (Fig. 2C). Since Fab311 is derived from a volunteer immunized with RTS,S and MGG4 is derived from a volunteer immunized with irradiated sporozoites, the similarities between the two imply that the previously reported potent public antibody lineage, from which MGG4 originates (*17*), can be accessed using the RTS,S vaccine candidate. This intriguing cryo-EM structure may provide the basis for design of previously unanticipated novel immunogens that now can take into account the 3D spiral architecture of the CSP repeat region rather than information derived solely from Fab-peptide studies with smaller numbers of repeats. We note that, during initial review of our manuscript, another publication appeared that found a similar evolution of Fab-Fab interactions to CSP in antibodies derived from patients immunized with whole attenuated sporozoites (*33*).

## MATERIALS AND METHODS

### Mutagenesis of the Fab 311 interface

The Fab311 light-chain mutations (R93S and R94S) were generated using QuikChange Site-Directed Mutagenesis (Agilent). The Fab311 heavy-chain mutations (N31S, R56N, N57K, and E64K) were generated using the In-Fusion HD Cloning Kit (Clontech).

### Isothermal titration calorimetry

ITC measurements were performed using a MicroCal Auto-iTC200 (GE Healthcare). Fab311 and Fab311R samples were dialyzed against Dulbecco’s phosphate-buffered saline (PBS) (Thermo Fisher Scientific). The Ac-(NPNA)_2_-NH_2_ peptide was purchased from InnoPep Inc. (>98% purity, chlorine counter ions) and dissolved in PBS. The peptide concentration in the syringe was 134 μM, and the Fab concentration in the cell was 9.2 and 9.6 μM for Fab311 and Fab311R, respectively. Concentrations were determined by ultraviolet absorbance at 280 and 205 nm for Fab and peptide, respectively. Triplicate experiments were carried out at 25°C and consisted of 16 injections, with a volume of 2.45 μl, a duration of 4.9 s, an injection interval of 180 s, and a reference power of 5 μcal. Origin 7.0 software was used to fit the data, where the first point and any outliers were excluded.

### Sample preparation

All Fabs and IgGs were produced in FreeStyle 293-F cells (Invitrogen). Fab311 and Fab317 were purified as previously described (*15*). IgG311 was purified using a HiTrap Protein G HP column (GE Healthcare). The rsCSP and flCSP were expressed and purified as previously described (*15*, *34*). Samples for cryo-EM and negative stain were made by mixing rsCSP or flCSP with 10- to 20-fold molar excess of IgG or Fabs and incubated overnight. The complexes were purified using a Superpose 6 increase 10/300GL gel filtration column (GE Healthcare) for the flCSP-Fab311 and rsCSP-IgG311 complexes and a Superdex 200 16/60 gel filtration column (GE Healthcare) for the rsCSP-Fab311 and rsCSP-Fab317 complexes, which were equilibrated with tris-buffered saline (TBS) buffer [50 mM tris-HCl, 137 mM NaCl, and 2.7 mM KCl (pH 8.0)].

### Negative-stain electron microscopy

Specimens were diluted to ~0.01 mg/ml with TBS buffer and deposited on glow-discharged copper mesh grids. Uranyl formate (2%) was placed on the grid for 30 s, which yielded uniform stain. Datasets were collected at ×52,000 magnification on a 120-keV Thermo Fisher Tecnai Spirit paired with a Tietz TVIPS CMOS 4k by 4k camera, with a −1.5-μm defocus value and a dose of 25 e^−^/Å^2^. The resulting pixel size was 2.05 Å per pixel. The Leginon software (*35*) automated the data collection process, and raw micrographs were stored in the Appion database (*36*). Particles were picked with DoGpicker (*37*), stacked with the box size of 224 pixels, and 2D classified with Iterative Multivariate statistical analysis (MSA) and Multi-reference alignment (MRA) (*38*). Particles of interest were selected for and exported into RELION (*39*) for another round of 2D and 3D classification/refinement. For all 3D classifications, the initial model was a 30-Å low-pass–filtered map of rsCSP and the variable regions of Fab311 from the cryo-EM model. All nsEM refinements in this study were subject to C1 symmetry.

### Sample vitrification for cryo-EM

The rsCSP-Fab311 and rsCSP-Fab317 complexes, each at a concentration of 5 mg/ml, were frozen with *n*-dodecyl-β-d-maltopyranoside to aid in sample dispersal. Each complex and detergent were briefly incubated together and then deposited on a Solarus plasma cleaned C-flat 2/2-4C grid (Protochips) and subsequently plunge frozen using a Thermo Fisher Vitrobot Mark IV. The settings for the Vitrobot were as follows: 4°C, 100% humidity, 10-s wait time, 4.5-s blot time, and a blot force of 0.

### Cryo-EM data collection

Data acquisition for rsCSP-Fab311 was facilitated with the Leginon software and a 300-keV Thermo Fisher Titan Krios paired with a 4k by 4k K2 Summit direct electron detector camera (Gatan). A total of 1497 micrographs were collected at ×29,000 magnification, with a total dose of 62 e^−^/Å^2^, which was fractionated over 48 raw frames, each receiving a dose rate of 5.5 electrons per pixel per second. The micrographs were collected with a defocus range of −0.5 to −2.5 μm and a pixel size of 1.03 Å.

For the rsCSP-Fab317 complex, a dataset of 470 micrographs at ×36,000 magnification was collected on a 200-keV Thermo Fisher Talos Arctica and a 4k by 4k Gatan K2 Summit direct electron detector camera, with a pixel size of 1.15 Å. Data acquisition was automated with Leginon, and micrographs were stored in Appion. Each micrograph received a dose rate of 5.6 electrons per pixel per second, for a total dose of 51 e^−^/Å^2^ fractionated over 48 raw frames. A defocus range of −0.5 to −2.5 μm was used.

### Cryo-EM data processing, model building, and refinement

Micrograph movie frames were aligned and dose weighted using MotionCor2 (*40*), while the contrast transfer function (CTF) was estimated using GCTF (*41*). An initial round of manual particle picking was done on 10 micrograph exemplars in Appion, which later served as a template for RELION. RELION template picked particles on dose-weighted micrographs that were subsequently extracted with a box size of 288 pixels. Particles were then imported into cryoSPARC (*42*), where reference-free 2D classification, initial model-free 3D classification, and 3D refinement were performed with no imposed symmetry. UCSF Chimera (*43*) was used to dock 11 copies of the Fab311-(NPNA)_3_ crystal structure (*13*) into the initial 4.7-Å 3D reconstruction of rsCSP-Fab311 generated in cryoSPARC and saved as a single model. The rsCSP peptide was stitched together in COOT (*44*), and the Fab constant regions of 311 were removed to generate an initial atomic model, which was then refined into the EM density map using RosettaRelax (*45*). The resulting atomic model was used to generate a 15-Å-resolution simulated EM density of the core rsCSP-Fab311, from which a volumetric mask was generated using RELION. The particle orientations and 3D reconstruction of rsCSP-Fab311 were exported from cryoSPARC into RELION for a final round of masked local 3D refinement and postprocessing, resulting in a ~3.37-Å-resolution map, sharpened with a *B* factor of −123 Å^2^. The rsCSP-Fab311 atomic model was further refined into the EM density map using RosettaRelax. A local resolution map was created using RELION. MS (*46*) was used to calculate BSAs using a 1.7-Å probe radius and standard van der Waals radii (*47*). Hydrogen bonds and salt bridges were evaluated using HBPLUS (*48*) and CONTACSYM (*49*). For rsCSP-Fab317, the particles did not converge to high resolution and, therefore, a model was not built.

### rsCSP-IgG311 modeling

Two copies of the Fab from the Fab311-(NPNA)_3_ crystal structure were docked onto the cryo-EM model of rsCSP-Fab311 using UCSF Chimera. The heavy-chain hinge region was extended manually using UCSF Chimera such that the Fc plus partial hinge region crystal structure 5v4e could be docked onto it. The torsion angles in the hinge region were adjusted manually in COOT such that all of the disulfides were appropriately satisfied. The resulting IgG model was idealized and relaxed using Rosetta (*45*). Five copies of the IgG model were docked onto the cryo-EM model of rsCSP-Fab311 using UCSF Chimera to generate the model shown in Fig. 5C.

## Supplementary Material

http://advances.sciencemag.org/cgi/content/full/4/10/eaau8529/DC1

## SUPPLEMENTARY MATERIALS

Supplementary material for this article is available at http://advances.sciencemag.org/cgi/content/full/4/10/eaau8529/DC1 Fig. S1. Flowchart of the data collection and processing pipeline that resulted in the final rsCSP-Fab311 cryo-EM structure. Fig. S2. Cryo-EM of the rsCSP-Fab311 complex. Fig. S3. Cryo-EM of the rsCSP-Fab317 complex. Fig. S4. Affinity measurements for Fab311 and Fab311R. Fig. S5. nsEM of the flCSP-Fab311 and rsCSP-IgG311 complexes. Fig. S6. Stoichiometry analysis of the flCSP-Fab311 and rsCSP-Fab311 complexes. Table S1. Cryo-EM data collection and processing statistics. Table S2. Isothermal titration calorimetry.
