# Supplementary material for: Cryo-EM structure of P. falciparum circumsporozoite protein with a vaccine-elicited antibody is stabilized by somatically mutated inter-Fab contacts
Source: Sci Adv. 2018 Oct 10;4(10):eaau8529. doi: 10.1126/sciadv.aau8529 (PMC6179375; doi:10.1126/sciadv.aau8529)
Supplement: http://advances.sciencemag.org/cgi/content/full/4/10/eaau8529/DC1 [file supp_4_10_eaau8529__index.html]

Science Advances | Science Advances

## Supplementary Materials

**This PDF file includes:**

- Fig. S1. Flowchart of the data collection and processing pipeline that resulted in the final rsCSP-Fab311 cryo-EM structure.
- Fig. S2. Cryo-EM of the rsCSP-Fab311 complex.
- Fig. S3. Cryo-EM of the rsCSP-Fab317 complex.
- Fig. S4. Affinity measurements for Fab311 and Fab311R.
- Fig. S5. nsEM of the flCSP-Fab311 and rsCSP-IgG311 complexes.
- Fig. S6. Stoichiometry analysis of the flCSP-Fab311 and rsCSP-Fab311 complexes.
- Table S1. Cryo-EM data collection and processing statistics.
- Table S2. Isothermal titration calorimetry.

Download PDF

**Files in this Data Supplement:**

- Adobe PDF - aau8529\_SM.pdf
