# Supplementary material for: Cryo-EM structure of P. falciparum circumsporozoite protein with a vaccine-elicited antibody is stabilized by somatically mutated inter-Fab contacts
Source: Sci Adv. 2018 Oct 10;4(10):eaau8529. doi: 10.1126/sciadv.aau8529 (PMC6179375; doi:10.1126/sciadv.aau8529)
Supplement: http://advances.sciencemag.org/cgi/content/full/4/10/eaau8529/DC1 [file aau8529_SM.pdf]

Science Advances
AAAS

advances.sciencemag.org/cgi/content/full/4/10/eaau8529/DC1

Supplementary Materials for

Cryo-EM structure of *P. falciparum* circumsporozoite protein with a vaccine-elicited antibody is stabilized by somatically mutated inter-Fab contacts

David Oyen, Jonathan L. Torres, Christopher A. Cottrell, C. Richter King, Ian A. Wilson*, Andrew B. Ward*

*Corresponding author. Email: wilson@scripps.edu (I.A.W.); andrew@scripps.edu (A.B.W.)

Published 10 October 2018, *Sci. Adv.* **4**, eaau8529 (2018)
DOI: 10.1126/sciadv.aau8529

This PDF file includes:

Fig. S1. Flowchart of the data collection and processing pipeline that resulted in the final rsCSP-Fab311 cryo-EM structure.
Fig. S2. Cryo-EM of the rsCSP-Fab311 complex.
Fig. S3. Cryo-EM of the rsCSP-Fab317 complex.
Fig. S4. Affinity measurements for Fab311 and Fab311R.
Fig. S5. nsEM of the flCSP-Fab311 and rsCSP-IgG311 complexes.
Fig. S6. Stoichiometry analysis of the flCSP-Fab311 and rsCSP-Fab311 complexes.
Table S1. Cryo-EM data collection and processing statistics.
Table S2. Isothermal titration calorimetry.

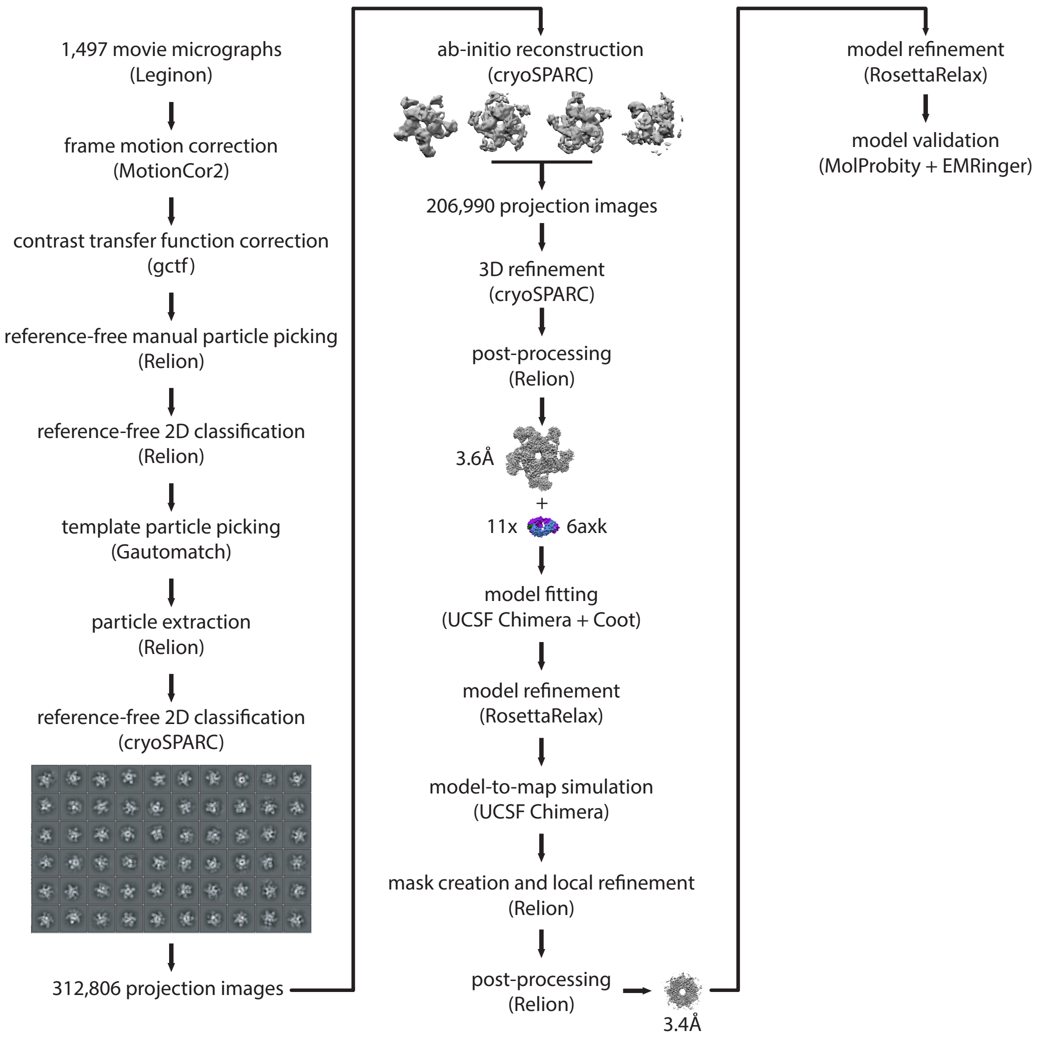

Fig. S1. Flowchart of the data collection and processing pipeline that resulted in the final rsCSP-Fab311 cryo-EM structure.

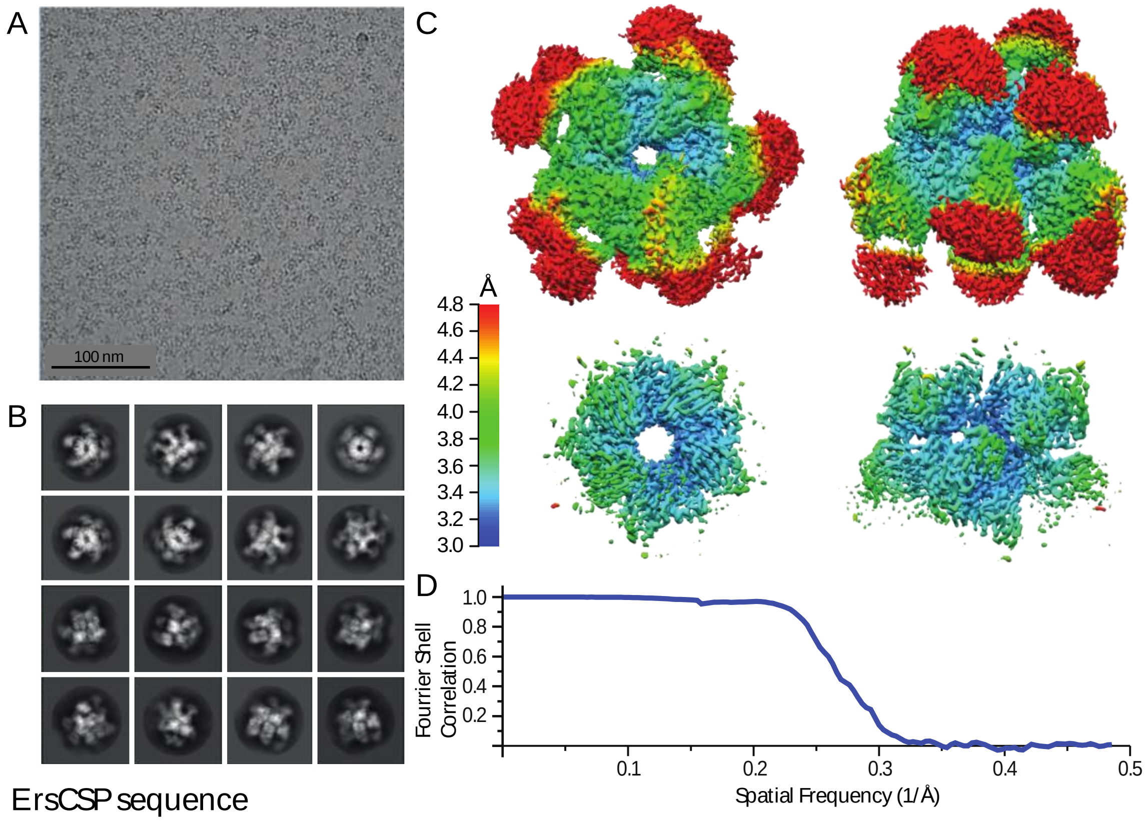

E rsCSP sequence

26 YGSSSNTRVLNELNYDNAGTNLYNELEMNYYGKQENWYSLKKNSRSLGENDDGNNEDNEKLRKPKHKKL
KQPADGNP DPNANPNVDPNANPNVDPNANPNVDPNANPNANPNANPNANPNANPNANPNANPNANPNAN
PNANPNANPNANPNANPNANPNANPNANPNKNNQGNGQGHNMPNDPNRNVDENANANSAVKNNNNEEPS
DKHIKEYLNKIQNSLSTEWSPCSVTCGNGIQVRIKPGSANKPKDELDYANDIEKKICKMEKCSSVFNVV
NS 303

Fig. S2. Cryo-EM of the rsCSP-Fab311 complex. Exemplar cryo-EM raw micrograph showing a high density of well-distributed rsCSP-Fab311 complexes (**A**). 2D class averages reveal many different orientations of the complex with Fabs clearly visible (**B**). Local resolution map, contoured at two different levels, demonstrates that the Fab variable regions and rsCSP are resolved at ~3 Å resolution, while the Fab constant domains are much lower resolution (**C**). Fourier shell correlation curve for the final reconstruction of the rsCSP-Fab311 complex (**D**). Sequence of the rsCSP construct, with the N- and C-terminal His tags removed for clarity (**E**). The Fab311 epitopes are colored alternating blue and red. A total of 11 epitopes are available for Fab311 binding.

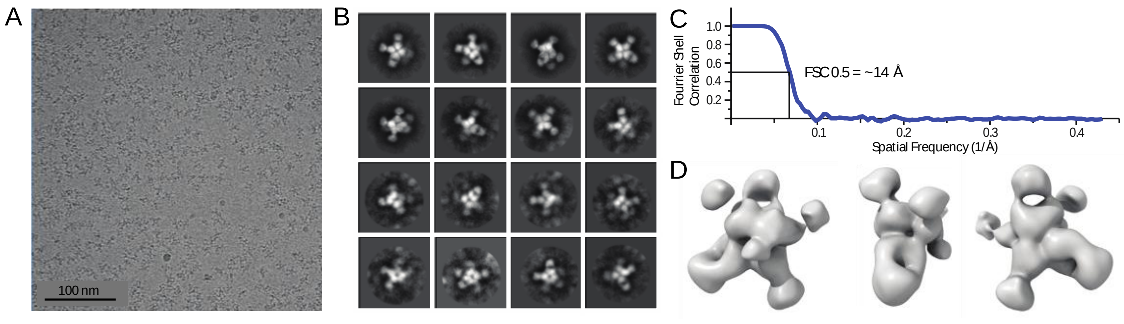

Fig. S3. Cryo-EM of the rsCSP-Fab317 complex. Exemplar cryo-EM micrograph (**A**), select 2D class averages (**B**), Fourier shell correlation curve (**C**), and three different views of the rsCSP-Fab317 reconstruction (**D**). The calculated resolution for the cryo-EM map is ~14 Å, using the 0.5 FSC cut-off. Notably, the reconstruction did not refine to high resolution, consistent with flexibility in the complex.

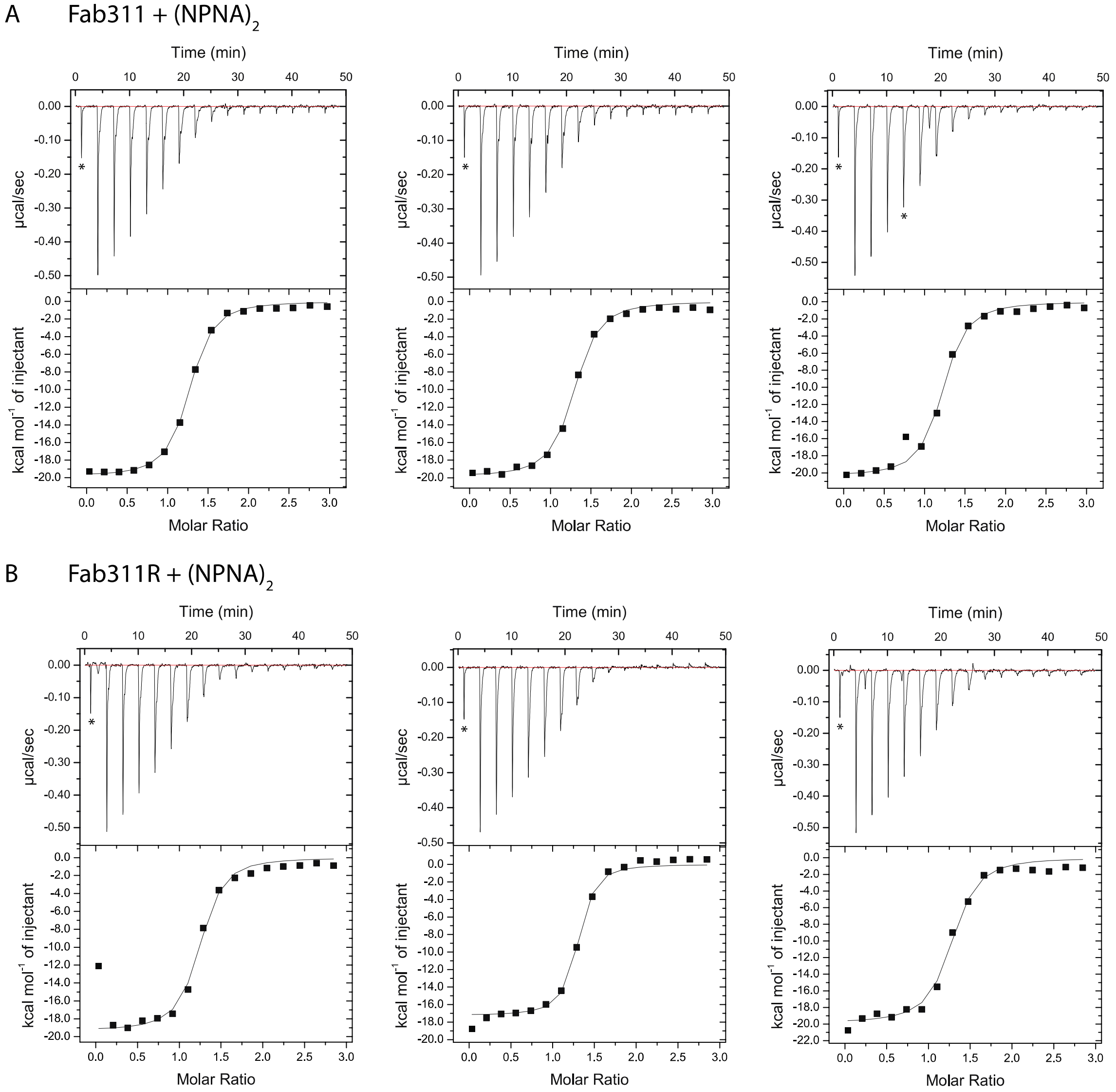

Fig. S4. Affinity measurements for Fab311 and Fab311R. Isothermal Titration Calorimetry binding data for Fab311 and Fab311R to the $(NPNA)_2$ peptide. Data points excluded from the fit are indicated with an asterisk.

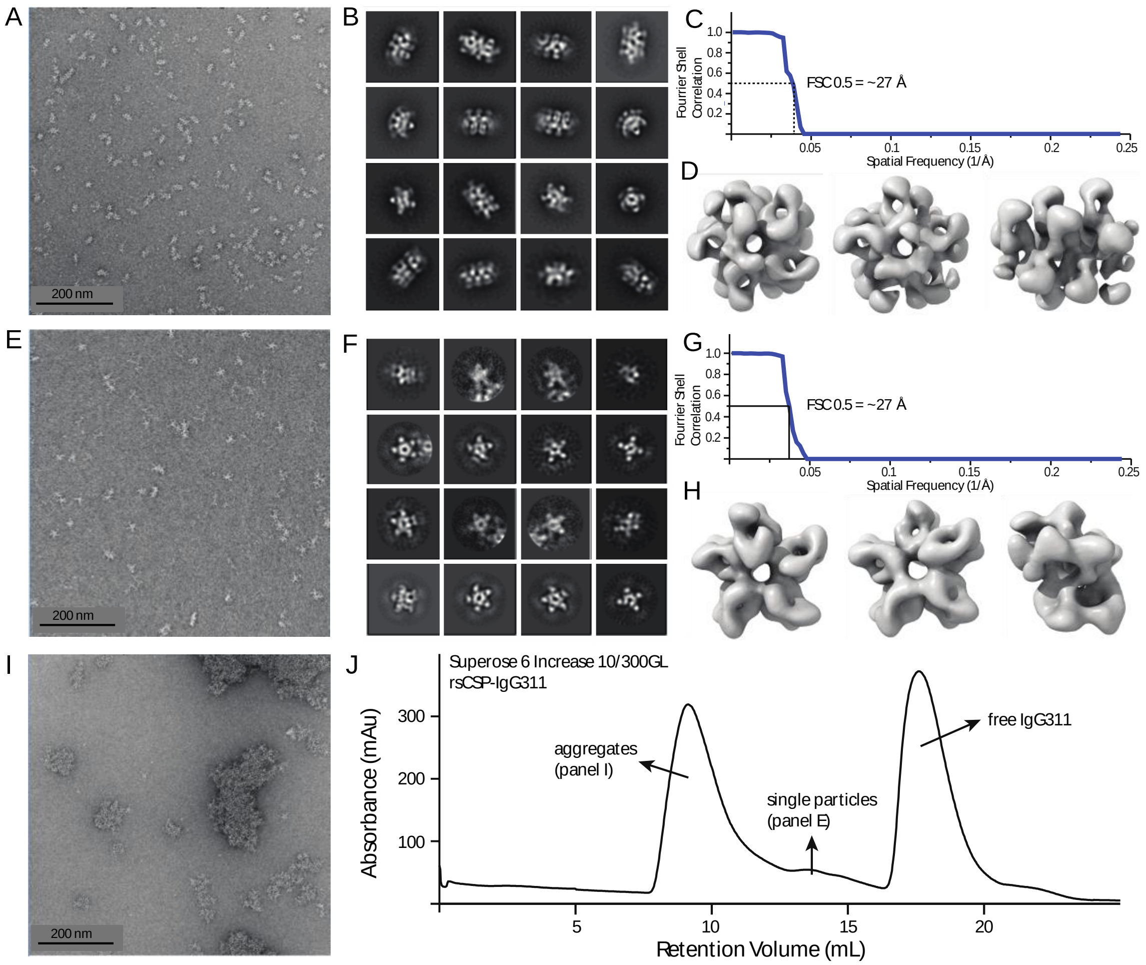

Fig. S5. nsEM of the flCSP-Fab311 and rsCSP-IgG311 complexes. Exemplar nsEM micrographs, select 2D class averages, Fourier shell correlation curves, and three distinct views of the refined nsEM maps of flCSP-Fab311(**A, B, C, D**), rsCSP-IgG311 (**E, F, G, H**) and the rsCSP-IgG311 aggregates (**I**). The calculated resolution for both maps is ~27 Å. (**J**) SEC chromatogram for the rsCSP-IgG311 complex.

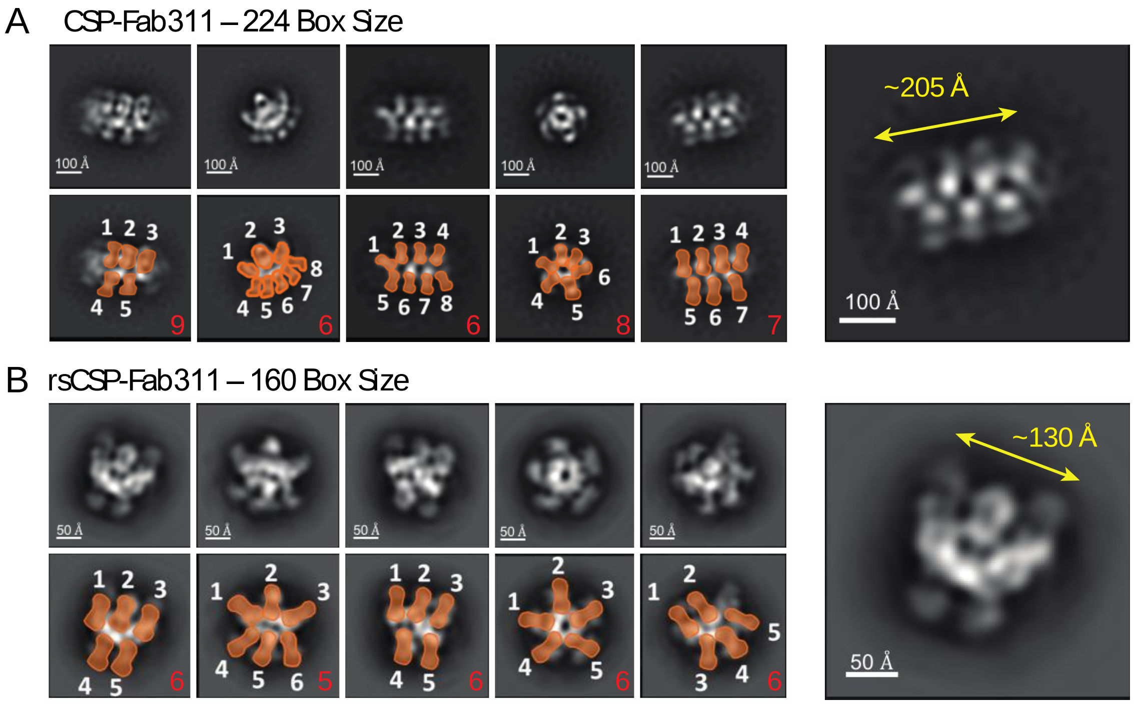

Fig. S6. Stoichiometry analysis of the flCSP-Fab311 and rsCSP-Fab311 complexes. Five representative class averages of flCSP+Fab311 from nsEM (**A**) and five matching back projections of the cryo-EM map of rsCSP+Fab311, low pass filtered to 20 Å (**B**). Fab311 is false colored in orange and Fabs visible in the back projection are enumerated in white. The number of eclipsed Fabs is listed in the right bottom corner of each class average in red, calculated by subtracting the total number of Fabs seen in the 3D reconstruction of the complex (14 for the flCSP-Fab311 and 10 for the rsCSP-IgG311), from the number of discernable Fabs in the class average (C). The estimated length based on the 3D reconstructions of flCSP+Fab311 is 205 Å (D) versus 130 Å for rsCSP+Fab311 (E).

Table S1. Cryo-EM data collection and processing statistics.

	Full rsCSP-Fab311	Masked rsCSP-Fab311
**Microscope**	FEI Titan Krios	
**Voltage (kV)**	300	
**Detector**	Gatan K2 Summit	
**Recording Mode**	Counting	
**Magnification**	29,000x	
**Movie Micrograph Pixel Size (Å)**	1.03	
**Dose Rate (e-/camera pixel/s)**	5.5	
**No. of Frames per Movie Micrograph**	48	
**Frame Exposure Time (ms)**	200	
**Movie Micrograph Exposure Time (s)**	9.6	
**Total Dose (e-/Å^2)**	62	
**Defocus Range (μm)**	0.5-2.5	
**No. of Movie Micrographs**	1,497	1,497
**No. of Molecular Projection Images in Map**	206,990	206,990
**Symmetry**	C1	C1
**Map Resolution (FSC=0.143)**	3.57	3.37
**Map Sharpening B-factor (Å^2)**	-88.2	-122.6
**No. of Atoms in Deposited Model**	19,931	16,423
**No. of Fabs in Deposited Model**	11	9
**MolProbity Score**	1.23	0.87
**Clashscore**	1.73	0.28
**EMRinger Score**	3.50	4.52
**EMDB**	9114	9065
**PDB ID**	6MHG	6MB3

Table S2. Isothermal titration calorimetry.

	Fab311-(NPNA)$_2$	Fab311R-(NPNA)$_2$
**N**	1.19 ± 0.04	1.21 ± 0.03
**K_d (nM)**	160 ± 7	135 ± 56
**ΔH (cal/mol)**	-20000 ± 289	-18800 ± 1393
**ΔS (cal/mol/degree)**	-36 ± 1	-32 ± 6
